# The Chemokine Receptor CXCR4 in Cell Proliferation and Tissue Regeneration

**DOI:** 10.3389/fimmu.2020.02109

**Published:** 2020-08-28

**Authors:** Marco E. Bianchi, Rosanna Mezzapelle

**Affiliations:** ^1^Division of Genetics and Cell Biology, IRCCS San Raffaele Scientific Institute, Milan, Italy; ^2^Vita-Salute San Raffaele University, Milan, Italy

**Keywords:** chemokine, cancer, tissue regeneration, CXCL12, HMGB1, CXCR4

## Abstract

The CXCR4 receptor upon binding its ligands triggers multiple signaling pathways that orchestrate cell migration, hematopoiesis and cell homing, and retention in the bone marrow. However, CXCR4 also directly controls cell proliferation of non-hematopoietic cells. This review focuses on recent reports pointing to its pivotal role in tissue regeneration and stem cell activation, and discusses the connection to the known role of CXCR4 in promoting tumor growth. The mechanisms may be similar in all cases, since regeneration often recapitulates developmental processes, and cancer often exploits developmental pathways. Moreover, cell migration and cell proliferation appear to be downstream of the same signaling pathways. A deeper understanding of the complex signaling originating from CXCR4 is needed to exploit the opportunities to repair damaged organs safely and effectively.

## Introduction

The binding of chemokines to G protein-coupled receptors (GPCRs) typically directs cell movement and traffic in and out of specific tissues in developing embryos and adult animals. They are also involved in tumor metastasis and invasion, and in the extension of neurites and axons of neurons (a part of a cell moves, while the cell body stays put). How chemokines and their receptors recruit hematopoietic cells to injured sites and tumors has been intensely investigated, whereas their involvement in the control of cell proliferation is less explored (1). Among chemokine receptors, CXCR4 is the most widely expressed, and is involved in numerous physiological and pathological conditions. CXCR4 is expressed by most cells, including hematopoietic and endothelial cells (ECs), neurons and stem cells (embryonic and adult). Increased levels of CXCR4 are present in cancer cells compared to the normal cells (2, 3). The focus of this mini-review is the emerging role of CXCR4 and its ligands in tissue repair and regeneration, and its relation to cancer cell proliferation. The role of CXCR4 in differentiation, retention, mobilization, migration, and polarization of hematopoietic cells is covered by other excellent reviews (4, 5).

## CXCR4 and its Ligands

CXCR4 is a 352 amino acid rhodopsin-like GPCR, comprising an extracellular N-terminal domain, 7 transmembrane (TM) helices, 3 extra-cellular loops (ECL), 3 intra-cellular loops (ICL) and an intracellular C-terminal domain (6). CXCR4 can exist in the plasma membrane as a monomer, dimer, higher-order oligomer or nanoclusters (7), although the partitioning and relevance of these different multimerization states has not been addressed *in vivo*. Several crystal structures of CXCR4 bound to agonists and small molecules are in accordance with the ability of CXCR4 to form homodimers via interactions of the TM5 and TM6 helices (6). TM6 is also implicated in nanoclustering (7). CXCR4 can also form heterodimers with ACKR3 (a related GCPR also known as CXCR7), which have distinctive signaling properties (8).

The canonical ligand of CXCR4 is CXCL12, also known as stromal cell-derived factor 1 (SDF-1) (9, 10). A single gene, CXCL12, codes for six protein isoforms in human (three in mouse), all deriving from alternative splicing of the fourth and final exon. The various forms are differentially expressed and have different affinities to glycosaminoglycans present on the cell surface and in the extracellular matrix (11). CXCL12α, an 89 amino acid protein, is the shorter and most expressed isoform (12, 13). Notably, CXCL12α can exist in monomeric and dimeric forms. CXCL12 only binds to chemokine receptors CXCR4 and ACKR3, itself a CXCR4 interactor; such a restricted receptor selectivity is unusual among chemokines.

The structure of the CXCR4/CXCL12 complex has not yet been determined; a model integrating homology modeling, experimentally derived restraints, and charge swap mutagenesis (14) highlights several contacts between the N-terminal tail of CXCR4 and CXCL12, and the interaction of the N-terminus of CXCL12 with the cavity delimited by the TM helices.

High mobility group box 1 protein (HMGB1) is the archetypal damage-associated molecular pattern (DAMP) molecule; DAMPs are released from dead or severely stressed cells to alert their microenvironment and the innate immune system. HMGB1 can form a heterocomplex with CXCL12 (HMGB1⋅CXL12) that also binds to CXCR4; of note, the conformational rearrangements of CXCR4 differ when triggered by CXCL12 alone or by HMGB1⋅CXCL12, and the complex is over one order of magnitude more potent than CXCL12 alone in inducing cell migration (15). Only the reduced form of HMGB1, where the pair of cysteines in the HMG-box domain A do not form a disulfide bond, binds CXCL12 and interacts with CXCR4 (16). However, a designer form of HMGB1 called 3S-HMGB1, where serines replace all three cysteines, binds to CXCR4 directly and is as effective as HMGB1⋅CXCL12 in promoting cell migration and muscle regeneration (17).

CXCR4 also binds macrophage migration inhibitory factor (MIF), a cytokine involved in the regulation of innate immunity (18). MIF binds to the N-terminal tail of CXCR4 and to the exterior side of TM helices, but not inside the TM pocket (18, 19). MIF also binds to other receptors, including CXCR2, CD74/CD44, and ACKR3 (20), which complicates the dissection of its activities.

Extracellular ubiquitin (eUb), also considered a DAMP, is a CXCL12 antagonist (21). Molecular modeling and mutagenesis suggest that it binds to CXCR4 inside the cavity delimited by TMs (22), but makes contact to CXCR4 residues that are not contributing to CXCL12 binding (23).

Beta-defensin-3 (HBD3) also competes with CXCL12 for CXCR4 binding, and promotes internalization of CXCR4 without inducing calcium flux, ERK phosphorylation, or chemotaxis (24).

Although the above list of actors is long, and multimeric complexes and multiple interactions increase complexity, genetics originated the widespread idea of CXCR4 and CXCL12 as a biunivocal couple: deletion in mice of either the *Cxcr4* or *Cxcl12* genes causes fetal lethality, defective B-cell lymphopoiesis, impaired bone-marrow myelopoiesis, and abnormal development of the cardiac septum and of the cerebellum (25, 26).

## CXCR4 and CXCL12 in Tissue Regeneration

Mice lacking CXCL12 or CXCR4 were first generated in the 1990s; since both die *in utero*, their ability to regenerate injured tissues was not investigated until later. Depletion of either CXCR4 or CXCL12 with small interfering RNAs injected in injured muscle impairs its regeneration, as does local injection of the CXCR4 antagonist AMD3100 (27), consistent with the expression of both CXCR4 and CXCL12 in skeletal muscle (28), and with impaired myogenesis and depletion of satellite cells in CXCR4 deficient mice (29). Satellite cells are the direct targets of CXCL12 (27).

More recently, CXCR4 and CXCL12 have been shown to control the regeneration of multiple organs and tissues, including lung, heart, liver, and the nervous system.

Surgical removal of one lung or part of it (pneumonectomy, PNX) is compensated by alveolar regrowth/regeneration in the remaining lung. After PNX, activated platelets trigger lung regeneration by binding to pulmonary capillary endothelial cells (PCECs) and supplying CXCL12 to activate CXCR4 and ACKR3 on their surface (30). PCECs activate AKT, proliferate and express the membrane metalloproteinase MMP14, which releases ligands that promote the proliferation of progenitor type II alveolar epithelial cells, and eventually alveolar regrowth. Endothelial cells are direct targets of CXCL12 via CXCR4, since genetic silencing of *Cxcr4* and *Ackr3* in PCECs impairs lung regeneration.

The mammalian heart cannot regenerate in adults, but it can in neonate mice (31). In myocardial infarction (MI), coronary arteries get obstructed, and must regenerate to support continued heart function. A unique CXCR4/CXCL12-dependent process termed “artery reassembly” allows the formation of an alternative (collateral) artery network to bypass obstructed or severed coronary arteries (32). In the mouse, within a few days after ligation of the left coronary artery on day 2 after birth, individual arterial endothelial cells (ECs) migrate out of the existing arteries, proliferate and then coalesce with capillaries, forming collateral arteries that connect branches of the right and left coronary arteries. A similar process reconnects severed arteries after the resection of the apex of the neonatal heart. Artery reassembly does not occur in adult hearts, but injection of a single dose of CXCL12 in the infarcted area promotes collateral formation and functional recovery of the heart. Notably, deletion of *Cxcl12* capillary ECs or *Cxcr4* in arterial ECs impairs artery reassembly; CXCL12 is not basally expressed in ECs, but hypoxia induces its expression. Thus, during artery reassembly different ECs are both source and target of CXCL12, via CXCR4.

Adult zebrafish hearts do regenerate, and coronary revascularization initiates within hours of injury. After cryoinjury, new coronaries regenerate both superficially around the injured area and intra-ventricularly toward the cardiac lumen, and act as a scaffold for proliferating cardiomyocytes (33). Epicardial cells express Cxcl12b after injury, as a consequence of hypoxia and HIF-1α activation. ECs in both superficial and intra-ventricular coronaries have a common origin and both express CXCR4, but inhibiting CXCR4 pharmacologically or deleting *Cxcr4* in the whole heart limits superficial, and not intra-ventricular, regeneration.

The liver is capable of continuous turnover and regeneration, which is overridden by fibrosis, cirrhosis and hepatic failure only after chronic or overwhelming injury. CXCL12 is constitutively expressed in healthy liver, and its expression increases following acute or chronic injury. Liver sinusoidal endothelial cells (LSEC) and hepatic stellate cells (HSC) are important sources of CXCL12 in liver disease. HSC and mesenchymal stem cells mainly respond via CXCR4, while LSEC express both CXCR4 and ACKR3. CXCL12 can activate HSC and recruit bone marrow mesenchymal cells, which promote liver fibrosis; in LSEC, CXCL12 signals via the physical association of CXCR4 and ACKR3 to activate eventually the transcription factor Id1, which orchestrates pro-regenerative responses, such as production of Wnt2 and hepatocyte growth factor (HGF) (34). Liver regeneration is abrogated by genetic silencing of either ACKR3 or CXCR4 in LSEC, or by chronic injuries that lead to excessive CXCR4 and reduced ACKR3 expression. *In vitro*, CXCL12 induces dose-dependent proliferation of human liver-derived stellate LX-2 cells, mediated by PI3K/Akt and Erk1/2 pathways (35).

The peripheral nervous system has retained throughout evolution the capability to regenerate. Recently, CXCL12 was found to promote the structural and functional recovery of the neuromuscular junction after degeneration of the motor axon terminal (36). CXCL12 is synthetized and released by peri-synaptic Schwann cells, and acts on CXCR4 re-expressed upon injury on the tip of the motor axon. CXCL12 also supports the functional and anatomical recovery of the sciatic nerve after crush injury; of special note, the small molecule NUCC-390, a CXCR4 agonist (37), also promotes nerve regeneration (38).

The central nervous system, in contrast, has a limited ability to regenerate, mostly dependent on neural progenitor cells (NPCs). Astrocytes are the main source of CXCL12 in the brain (39); CXCR4 is expressed on NPCs and CXCL12 appears to stimulate directly their *in vitro* proliferation and differentiation into neurons (40–42), via PI3K-Erk1/2 (43) and/or AKT/FOXO3α (44) activation. However, Li at al. (45) found no CXCL12-induced proliferation of NPC cells from E12 mouse embryos. CXCR4 activation by CXCL12 promotes the differentiation of human embryonic stem cells into neural stem cells (46) and then helps to maintain their stemness (47).

Overall, these studies implicate CXCR4 and CXCL12 in the regeneration of multiple organs, via CXCL12 release from various sources and CXCR4 activation on endothelial and progenitor cells, which then go on to proliferate; so far, a role of CXCR4 activation on parenchymal cells is not convincingly proven nor excluded. Hematopoietic and mesenchymal cells also contribute to tissue regeneration, but in this case the role played by the CXCL12/CXCR4 system appears limited to directing their chemotaxis to the damaged site.

## The HMGB1⋅CXCL12 Complex

The existence of the HMGB1⋅CXCL12 complex was first inferred from the ability of HMGB1 to promote the migration of endothelial, hematopoietic and mesenchymal cells (15) via CXCR4; the complex was then biochemically characterized (48). The complex was also found to promote the regeneration of skeletal muscle, since the reduced HMGB1 expression in *Hmgb1*+/− mice delays muscle regeneration (49), whereas the injection of exogenous reduced HMGB1 accelerates muscle, bone and liver repair in mouse (17, 50). Several cell-specific responses are involved, including the proliferation of satellite cells, skeletal stem cells and hepatocytes. The requirement for HMGB1, as opposed to CXCL12 alone, is supported by several observations: injection of CXCL12 alone promotes abnormal bone regeneration, with a larger fracture callus without a concomitant increase in bone mineral density and mechanical strength (50); local injection of glycyrrhizin, a HMGB1 inhibitor (51), delays bone fracture healing; injection of 3s-HMGB1, a mutant form of HMGB1 that can bind to CXCR4 in the absence of CXCL12, mimics the biological effects of HMGB1⋅CXCL12, including the promotion of *in vitro* myogenesis (17).

Remarkably, systemic injection of fully reduced HMGB1 (frHMGB1) or 3S-HMGB1 predisposes muscle and bone to regeneration/repair even if injected 2 weeks before injury (50), by inducing the transitioning of resting stem cells to a dynamic state of the cell cycle, intermediate between G_0_ and G_1_, termed “G_Alert_” (52). In contrast to deeply quiescent G_0_ stem cells, G_Alert_ stem cells are more metabolically active, contain higher levels of ATP and mitochondrial DNA, are larger and poised to enter the cell cycle when exposed to activating signals. Activation mTORC1 is both necessary and sufficient for the transitioning to the G_Alert_ state (53), and rapamycin, an mTORC inhibitor, interferes with HMGB1-induced transitioning to G_Alert_ (54). Multiple stem cell types (SSCs, satellite cells and hematopoietic stem cells) in mice subject to bone fracturing or muscle damage transition to the G_Alert_ state, and this requires HMGB1⋅CXCL12, since stem cells in HMGB1-deficient mice do not transition to the G_Alert_ state after injury unless exogenous HMGB1 is provided.

Thus, HMGB1⋅CXCL12 has similar activities to those reported for CXCL12 in muscle regeneration, but is absolutely required in G_Alert_ transitioning of stem cells. In this context, two questions arise: is HMGB1⋅CXCL12 (as opposed to CXCL12 alone) responsible for the regeneration of most or all tissues? Does HMGB1⋅CXCL12 also promote the proliferation of ECs? Indeed, HMGB1 has been shown to promote the proliferation of ECs of different origin, although the involvement of CXCR4 as the cognate receptor was not investigated (55).

## CXCR4/CXCL12 in Cancer Growth

Tumor is an illegitimate tissue that grows out of control because of an altered expression and behavior of pro-proliferative and pro-survival signals. Precisely because tumor tissue is out of balance with the surrounding legitimate tissues, it is also in a state of distress, similar to an injured tissue, and recruits inflammatory cells that support it. Famously, it has been said that a tumor is wound that never heals (56).

Chemokines and their receptors not only drive the trafficking of leukocytes inside the tumor mass but also contribute to most aspects of tumor cell biology (1). High expression of CXCR4 is observed in hematological malignancies (57–59) and in many types of solid tumors, including melanomas and kidney, lung, brain, prostate, breast, pancreas and ovarian tumors (2, 3), where it correlates with poor prognosis (59). Interestingly, the normal tissue adjacent to the CXCR4 overexpressing tumor shows normal or no CXCR4 expression (41), which suggests a differential response of cancer cells to microenvironmental conditions. Expression of CXCR4 and CXCL12 in cancer cells is also controlled by specific microRNAs: CXCL12 by miR-1 (60), miR-9 (61, 62), miR-126 (63), miR-146a (64), and miR-150 (65), whereas miR-200a can increase CXCR4 expression (66).

The expression of CXCR4/CXCL12 in tumors is partially dependent on the hypoxic tumor microenvironment, in a HIF-1α dependent manner (42). As a consequence of CXCL12 release, tumor-associated CXCR4-expressing ECs proliferate (67). CXCR4 is also expressed on putative cancer stem cells populations in various tumors, including renal (68), prostate (69) and non-small lung cancer (70), and affects their clonogenicity and spherogenicity, with adverse effects on prognosis. These CXCL12/CXCR4 effects are similar to the promotion of endothelial and stem cell proliferation in injured tissue.

Moreover, many reports indicate that binding of CXCL12 to CXCR4 on tumor cells of various types enhances their proliferation, both *in vitro* and *in vivo*, either via MAPK or PI3K/Akt pathways (54, 71, 72).

Table 1 lists a sample of reports on the role of CXCR4/CXCL12 in tumor cell proliferation (mostly tumor cell lines) (53, 69, 72–80). Targeting of CXCR4 with antibodies or specific inhibitors, most commonly AMD3100, has been intensely investigated; however, AMD3100/Plerixafor/Mozobil has been approved for bone marrow transplantation, but not as anti-cancer treatment.

**TABLE 1:** CXCR4/CXCL12 axis is involved in cancer cell proliferation.

**Tumor type**	**Cancer cell lines**	**Pathway involved**	**References**
Glioblastoma	Glioblastoma cell lines GB1690, 5GB, HTB-16	–	Sehgal et al. (72)
	Glioblastoma cell lines U87-MG, DBTRG-05MG	ERK; AKT	Barbero et al. (70)
Non-small cell lung cancer (NSCLC)	NSCLC cell lines L3, L4, A549	ERK	Wald et al. (73)
Malignant mesothelioma (MM)	MM cell lines H28, 211H, H2052, ms-1, H290, H513	AKT/mTOR	Li et al. (74)
Breast cancer	Breast cancer cell line MCF-7	–	Hall et al. (75)
Ovarian cancer	Ovarian cancer cell lines BG-1, SKOV3	–	Hall et al. (75), Guo et al. (76)
Colorectal cancer (CRC)	CRC cell lines HT-29, CaCo21, Colo320	PI3K/AKT	Ma et al. (77)
Pancreatic cancer	Pancreatic cancer cell lines AsPC-1, SW1990, BxPC-3	–	Gao et al. (78)
Esophageal squamous cell carcinoma (ESCC)	ESCC cell line EC9706 (*in vitro* and ESCC mouse xenograft model)	G0/G1 cell cycle arrest and apoptosis induction	Wang et al. (79)
Extrahepatic hilar cholangiocarcinoma (hilar-CCA)	Hilar-CCA cell line QBC939	–	Tan et al. (80)
Prostate cancer	Prostate cancer cell lines DU145 and PC3	PI3K/AKT	Dubrovska et al. (67)

*Many primary tumors overexpress CXCR4 and CXCL12 compared to their normal cells. The activation of CXCR4/CXCL12 in tumor cells leads to cell proliferation. –, not known.*

## CXCR4 Signaling

The preceding sections have highlighted that CXCR4 activation can drive both cell migration and cell proliferation, at least in vascular, progenitor and tumor cells. We will now review current information on the signaling involved.

**FIGURE 1:**
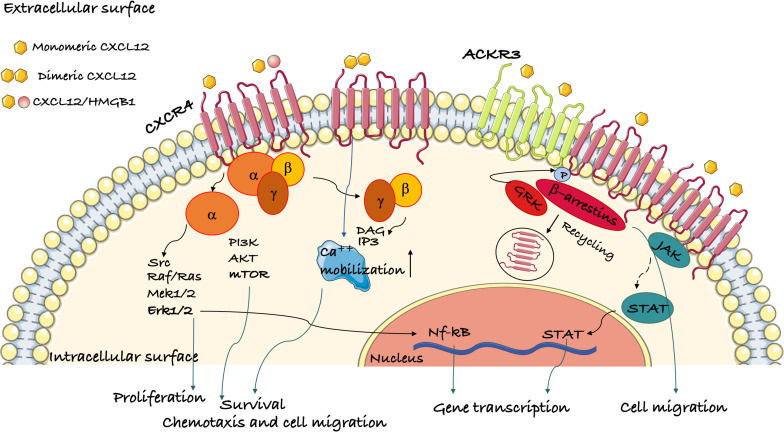
Schematic representation of the signaling pathways activated by CXCR4. Ligand binding to CXCR4 activates G protein subunits and the downstream Ca^2+^ mobilization from intracellular stores and PI3K/Akt, PLC, and ERK1/2 pathways. This results in gene transcription, cell migration, proliferation and survival. CXCR4 oligomerization can also activate the G-protein independent JAK/STAT pathway. β-arrestins are recruited following GRK phosphorylation of CXR4 and mediate its internalization. ACKR3 is another receptor for CXCL12 that can induce β-arrestin-mediated signaling both by itself or as a heterodimer with CXCR4.

Ligand binding to CXCR4 induces conformational changes that lead to the activation of multiple signaling pathways (Figure 1), originating proximally from the dissociation of heterotrimeric G proteins and from the phosphorylation of the C-terminal cytoplasmic tail of CXCR4. CXCR4 is mainly bound to heterotrimeric G_i_ proteins, although other G protein classes may transduce CXCR4 binding as well (81). Upon ligand binding, the G_i_ heterotrimer detaches from the CXCR4 intracellular loops and dissociates into GTP-bound α_i_ and βγ subunits (82, 83). The βγ subunits directly bind and activate phosphatidylinositol-3-OH kinases (PI3K) β or γ, which produce phosphatidylinositol triphosphate (PIP3), and phospholipase C β (PLC-β), which produces inositol-(1,4,5)-trisphosphate (IP3) and diacylglycerol (DAG). The Gα_i_ subunit induces calcium release from intracellular stores and indirectly activates the PI3K-AKT and MEK1/2-Erk1/2 axes (84). Via the production of PIP3, PI3Ks activate the serine-threonine kinase AKT, which can then can phosphorylate many target proteins, most notably glycogen synthase kinase 3 (GSK3), tuberous sclerosis 2 (TSC2), caspase 9 and PRAS40 (AKT1S1), which explains its wide spectrum of downstream effects in promoting cell proliferation, differentiation, apoptosis, angiogenesis, and metabolism (85).

CXCR4 ligand binding induces JAK/STAT activation in a Gα-independent manner (86). GPCR kinases (GRKs) phosphorylate multiple serines/threonines in the cytoplasmic tail of CXCR4. Phosphorylated CXCR4 recruits β-arrestin-1 and -2, which promote CXCR4 internalization (87). Thereafter, CXCR4 can be recycled back to the plasma membrane or sorted to the lysosomes for degradation (88). Of note, the recruitment of β-arrestins to CXCR4 also activates Erk signaling (89).

The binding of CXCL12 to CXCR4-ACKR3 heterodimers activates G protein-independent signaling cascades originating from β-arrestins that potentiate cell migration (8).

Overall, the activation of PI3Ks and Akt supports the proliferation and survival of both normal and cancer cells. mTORC activation underpins the anabolic metabolism that is required for cell growth; indeed, mTORC activation is also necessary for the transitioning of stem cells to the G_Alert_ state.

Notably, the CXCR4-activated pathways that direct cell movement and migration are exactly the same that are involved in cell proliferation, and both processes can be inhibited by the same small molecules. For example, rapamycin is an mTORC inhibitor that blocks cell proliferation, but it inhibits cell migration as well (90, 91). The same is true for PI3K inhibitors (92).

Although the various pathways originating from CXCR4 are known, there is ample scope for cell specificity. The human genome encodes 18 different Gα proteins, 5 Gβ proteins and 12 Gγ proteins, and multiple PI3Ks and PLCs, with ample variation of expression in different cell types. Moreover, signaling is enhanced or dampened by dozens of modulators, including scaffold proteins that facilitate the physical interactions of kinases and other enzymes that introduce post-translational modifications. We are unaware of studies that delineate the CXCR4-initiated signaling pathways in cell proliferation down to the specific isoforms and post-translational modifications of the signal transducers involved. Cancer is not the most amenable biological system, since cancer cells have accumulated a number of genetic and epigenetic alterations, often including those of PI3Ks. Cell-specific conditional mutants could be used to investigate CXCR4-controlled proliferation following injury, and this would provide a list of parts in specific cells; even so, we would still miss mechanistic details such as the interaction with modifiers, possible feed-forward and feedback loops and time-dependent signal adaptations like those involving Rac (93) or oscillatory behaviors like those described for NF-κB and p53 (94, 95).

## Conclusion

We have discussed several reports showing that CXCR4 can control cell proliferation in addition to directing cell retention and movement, both in physiological processes, such as development and tissue regeneration, and in pathological ones, such as cancer growth. The mechanisms and pathways involved may be broadly similar in all cases, since regeneration often recapitulates developmental processes, and cancer often exploits developmental pathways.

Signal transduction pathways downstream CXCR4 eventually control both cell movement and cell proliferation, which are both dependent on PI3K-Akt and mTORC signaling; the details, however, may vary from cell to cell and in different settings.

So far, the interest has focused on cancer and on drugs that block CXCR4-initiated signaling; we suggest that small molecules that activate CXCR4 signaling or can dissect the effects on cell migration and proliferation may be as useful.

## Author Contributions

Both authors contributed equally to the writing of the review.

## Conflict of Interest

The authors declare that the research was conducted in the absence of any commercial or financial relationships that could be construed as a potential conflict of interest.
